# Impaired extinction of learned fear in rats selectively bred for high anxiety – evidence of altered neuronal processing in prefrontal-amygdala pathways

**DOI:** 10.1111/j.1460-9568.2008.06511.x

**Published:** 2008-12

**Authors:** Patrik Muigg, Alfred Hetzenauer, Gabriele Hauer, Markus Hauschild, Stefano Gaburro, Elisabeth Frank, Rainer Landgraf, Nicolas Singewald

**Affiliations:** 1 Department of Pharmacology and Toxicology, Institute of Pharmacy, Center for Molecular Biosciences Innsbruck, University of InnsbruckPeter-Mayer-Strasse 1, A-6020 Innsbruck, Austria; 2 Department of Behavioral Neuroendocrinology, Max Planck Institute of PsychiatryMunich, Germany

**Keywords:** amygdala, c-Fos, cued fear conditioning, HAB, medial prefrontal cortex

## Abstract

The impaired extinction of acquired fear is a core symptom of anxiety disorders, such as post-traumatic stress disorder, phobias or panic disorder, and is known to be particularly resistant to existing pharmacotherapy. We provide here evidence that a similar relationship between trait anxiety and resistance to extinction of fear memory can be mimicked in a psychopathologic animal model. Wistar rat lines selectively bred for high (HAB) or low (LAB) anxiety-related behaviour were tested in a classical cued fear conditioning task utilizing freezing responses as a measure of fear. Fear acquisition was similar in both lines. In the extinction trial, however, HAB rats showed a marked deficit in the attenuation of freezing responses to repeated auditory conditioned stimulus presentations as compared with LAB rats, which exhibited rapid extinction. To gain information concerning the putatively altered neuronal processing associated with the differential behavioural response between HAB and LAB rats, c-Fos expression was investigated in the main prefrontal-amygdala pathways important for cued fear extinction. HAB compared to LAB rats showed an attenuated c-Fos response to repeated conditioned stimulus presentations in infralimbic and cingulate cortices, as well as in the lateral amygdala, but facilitated the c-Fos response in the medial part of the central amygdala. In conclusion, the present results support the notion that impaired extinction in high anxiety rats is accompanied by an aberrant activation profile in extinction-relevant prefrontal-amygdala circuits. Thus, HAB rats may represent a clinically relevant model to study the mechanisms and potential targets to accelerate delayed extinction processes in subjects with enhanced trait anxiety.

## Introduction

Impaired extinction of fear memories is a core feature in different anxiety disorders including phobias, post-traumatic stress disorders and panic (e.g. Jeffrey & Jay, 1998;Myers & Davis, 2002; Cammarota *et al.*, 2003; Quirk & Gehlert, 2003; Lissek *et al.*, 2005; Anderson & Insel, 2006). Currently a substantial proportion of anxiety patients do not respond effectively to the standard treatments, namely cognitive behavioural therapy and/or pharmacotherapy (Pull, 2007). For the development of more efficient drugs with the potential to enhance fear extinction processes, a better understanding of the underlying neurobiology is necessary.

Classical fear conditioning is a frequently used experimental model enabling the study of how the brain builds fear memories and how these memories come to be suppressed when they no longer predict danger (Barad, 2005; Maren, 2005). Behavioural and neural mechanisms of fear acquisition and expression are already well understood, thanks to animal and human studies (for review, see e.g. Fendt & Fanselow, 1999; Phelps & LeDoux, 2005). The neuronal mechanisms by which fear is inhibited are less well understood, although major progress has been made in this field in the last few years (for review, see Sotres-Bayon *et al.*, 2006; Myers & Davis, 2007; Quirk & Mueller, 2008). This is even more true for mechanisms underlying pathologically impaired fear extinction. We therefore attempted to investigate whether the extinction of conditioned fear is disturbed in a psychopathological animal model of enhanced trait anxiety, which mirrors important features of human anxiety pathology. There are a number of such animal models available, including models developed by selective breeding (for review, see Fujita *et al.*, 1994; Blizard & Adams, 2002; Landgraf & Wigger, 2002; Brush, 2003; Ramos *et al.*, 2003; Brunelli, 2005; Kromer *et al.*, 2005; Ohl, 2005; Steimer & Driscoll, 2005). We used Wistar rat lines selectively bred for high (HAB) or low (LAB) anxiety-related behaviour depending on their behaviour on the elevated plus maze (Liebsch *et al.*, 1998). HAB and LAB rats proved to be extremely divergent in innate, unconditioned anxiety as revealed in different behavioural tests (Landgraf *et al.*, 2007). Although a genetic relationship between anxiety-related behaviour and fear conditioning has been suggested (Ponder *et al.*, 2007), the latter has not yet been tested in HAB/LAB rats.

The specific aims of the present study were: (i) to investigate whether HAB rats compared with LAB rats show evidence of impaired extinction of conditioned fear and (ii) given that we find line differences in fear responding during the extinction training, to then use immediate early gene mapping to reveal potential line differences in the processing of neuronal activity in relevant fear-related brain areas. It has been repeatedly shown in rodents that auditory fear conditioning increases the products (both mRNA and protein) of the activity-dependent immediate early gene *c-fos* in brain regions related to the fear response (Pezzone *et al.*, 1992; Smith *et al.*, 1992; for review, see Knapska *et al.*, 2007). Thus, we decided to use c-Fos protein expression as an established marker of neuronal activation providing high (single cell) spatial resolution (Morgan & Curran, 1991; Hoffman & Lyo, 2002; Singewald, 2007). As we revealed in the first experiment that extinction of learned fear differed between the lines, we focused the c-Fos mapping on the amygdala and medial prefrontal cortex (mPFC) because these brain areas and their interactions have been shown to play a predominant role in fear extinction processes (for review, see Quirk *et al.*, 2006; Sotres-Bayon *et al.*, 2006; Akirav & Maroun, 2007). The hypothesis that we tested here was that differences in the c-Fos response in these key brain areas of extinction pathways should be found in rats that show delayed vs. rapid extinction of learned fear.

## Materials and methods

### Animals

All animals tested were bred in the animal facilities of the Max Plank Institute of Psychiatry (Munich, Germany) as described previously (Landgraf & Wigger, 2002). The experiments were performed using adult male HAB (*n*= 24) and LAB (*n*= 27) rats, aged 13–16 weeks and weighing 300–400 g at the time of experiments at 6 weeks after their arrival in Innsbruck. Rats were housed in groups of four to six per cage under standard laboratory conditions (12:12 h light/dark cycle with lights on at 07:00 h, 21°C, 50% humidity, pelleted food and water *ad libitum*). Procedures were approved by the national Ethical Committee on Animal Care and Use (Bundesministerium für Wissenschaft und Verkehr, Kommission für Tierversuchsangelegenheiten, Austria).

### Cued auditory fear conditioning

Two different contexts, A and B, were used for the fear conditioning experiments. A fear conditioning chamber (26 × 30 × 32 cm; Coulbourn Instruments, Allentown, PA, USA) served as context A and a standard empty rat cage (type 3 cage; 22 × 37.5 × 15 cm) served as context B. In order to maximally reduce the contribution of context to cued fear conditioning, tactile, visual and olfactory cues were different in context B compared with context A. Thus, context A was equipped with a metal grid, the illumination was bright light (300 lx) and the chamber was cleaned with water after each use. Context B was a cage with a smooth surface, illumination was dim red light (∼10–15 lx) and the cage was wiped out with ethanol after each session. Individual video cameras were mounted above each context and connected to a video recorder. Freezing behaviour, used as a measure of fear (e.g. Maren, 2005; Phelps & LeDoux, 2005), was defined as the absence of all non-respiratory movements (Fanselow, 1980) and was rated from a videotape by an experienced investigator blind to the groups. Auditory stimuli (see below) for the cued fear conditioning task were delivered via a speaker (Coulbourn Instruments) mounted approximately 20 cm above the contexts. Unconditioned stimuli (USs) were delivered via an interface connected to the metal grid of context A.

### Experiment 1: behaviour of HAB and LAB rats during acquisition and extinction of conditioned fear

#### Day 1

For acquisition, single-housed HAB and LAB rats were transferred in their home cage from the animal facilities directly to the experimental room, placed in context A and habituated in the acquisition chamber for 5 min. Fear acquisition was elicited by presenting audible cues [conditioned stimulus (CS); white noise, 80 dB, 30 s, five times] that co-terminated with mild, short foot shocks (US; 0.7 mA, 2 s). Stimulus-free periods (2 min) preceded, separated and followed the pairings. Following the CS/US pairing, rats were left in the acquisition chamber for 5 min before they were transferred back to the animal facilities.

#### Day 2 and 3

Extinction was carried out in context B after 24 h of memory consolidation. Rats received 30 CS presentations (white noise, 30 s, 80 dB, 5 s inter-stimulus interval) at 5 min after placement in context B. Rats were returned to their home cages at 5 min after presentation of the final CS. Extinction recall on day 3 was performed in context B at 24 h after extinction training in a separate set of HAB (*n*= 9) and LAB (*n*= 10) rats. After a habituation time of 5 min, HAB and LAB rats received one CS presentation (white noise, 30 s, 80 dB). After extinction recall, animals were returned to their home cage. Animals that were fear conditioned on day 1 [C(+)] and exposed to the extinction session on day 2 were termed C(+)CS in Experiment 2 (see below).

#### Faecal boli quantification

As an additional, indirect indicator of emotionality (Hall, 1934; Royce, 1977; Sarbadhikari *et al.*, 1996), the number of faecal boli shed during acquisition and extinction trials was quantified.

### Experiment 2: c-Fos expression pattern in HAB and LAB rats in response to the fear extinction trial

**Fig. 2:**
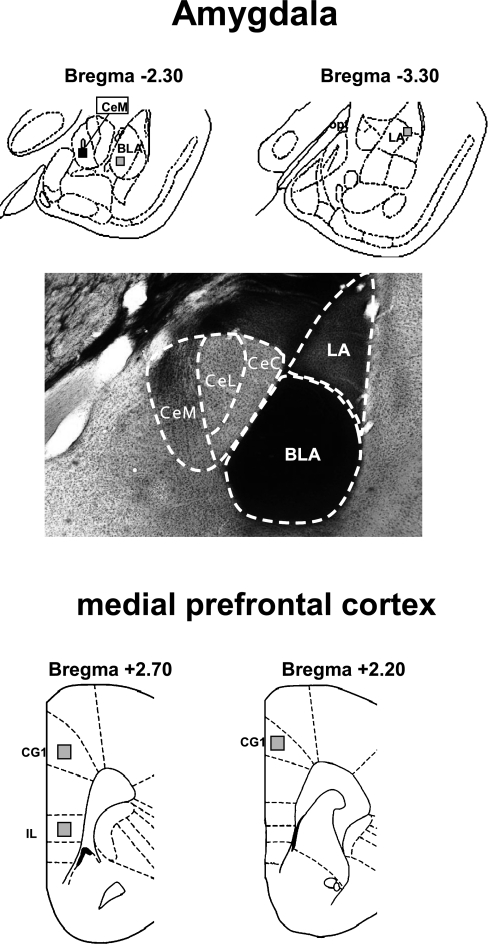
Schematic diagrams adapted from the atlas of Paxinos & Watson (1998) showing subregions of the amygdala and mPFC in which a differential c-Fos response after repeated conditioned auditory stimulus presentation in the extinction trial was found in HAB vs. LAB rats [C(+)CS groups]. Acetylcholinesterase staining was performed to aid identification of CeA subdivisions. Grey boxes indicate reduced c-Fos expression in HAB vs. LAB rats and black box indicates increased c-Fos expression in HAB vs. LAB rats. CeC, capsular part of the central nucleus of the amygdala; CeL, lateral part of the central nucleus of the amygdala; opt, optic nerve.

A second set of group-housed HAB and LAB animals was used to investigate whether the altered extinction of learned fear observed in single-housed HAB rats (Experiment 1) would be replicated in group-housed animals and whether HAB rats as compared with rapidly extinguishing LAB rats would show differential activation patterns (assessed by c-Fos mapping) in fear extinction-related brain areas. For the c-Fos study we included two control groups in addition to the C(+)CS extinction group (see also above). Rats of the C(+)no-CS group were fear conditioned on day 1 and placed into context B on day 2 but received no CS. Rats of the second control group [C(−)CS] received five CS only presentations on the first day and 30 CS presentations on the second day. These controls were included in order to test if repeated presentation of CS (without prior aversive conditioning) affects the c-Fos pattern in HAB and LAB rats. Rats of the extinction group and the two control groups were kept in context B for the same time period (22.5 min). c-Fos expression elicited under these conditions was then investigated in cortico-limbic areas known to be particularly relevant for extinction processes (see Introduction). Detailed quantification was performed in subregions of the amygdala at five different levels (Bregma −1.80, −2.30, −2.80, −3.30 and −3.60) and in the mPFC at two different levels (Bregma +2.70 and +2.20) (Table 1, Fig. 2).

**Table 1:** c-Fos expression in medial prefrontal cortical and amygdaloid regions following the within-session extinction trial in HAB and LAB rats

	Control groups				Extinction groups		
	C(+)no-CS		C(−)CS		C(+)CS		
Brain regions and brain levels	HAB	LAB	HAB	LAB	HAB	LAB	HAB vs. LAB
Amygdala nuclei							
Bregma −1.80							
Central, medial	1.3 ± 0.2	1.4 ± 0.3	0.8 ± 0.2	1.5 ± 0.3	1.1 ± 0.1	1.3 ± 0.3	
Central, lateral	1.0 ± 0.4	1.2 ± 0.3	1.0 ± 0.3	1.3 ± 0.3	1.1 ± 0.1	1.3 ± 0.3	
Central, capsular	1.2 ± 0.2	1.3 ± 0.2	1.0 ± 0.3	1.8 ± 0.8	2.3 ± 0.4	2.2 ± 0.4	
Basolateral, anterior	1.3 ± 0.4	1.1 ± 0.4	1.5 ± 0.3	1.3 ± 0.2	2.9 ± 0.6	3.4 ± 0.5	
Medial, anterodorsal	5.8 ± 0.1	7.8 ± 0.7	3.7 ± 0.7	6.8 ± 0.8	6.5 ± 1.1	7.4 ± 0.8	
Basomedial, anterior	2.2 ± 0.4	4.0 ± 0.7	2.0 ± 0.5	3.2 ± 0.7	3.8 ± 0.5	4.9 ± 0.4	
Cortical, anterior	5.8 ± 1.0	7.4 ± 0.9	3.3 ± 1.2	5.7 ± 1.4	6.9 ± 0.8	8.8 ± 0.9	
Bregma −2.30							
Central, medial	0.1 ± 0.1	0.5 ± 0.4	0.5 ± 0.0	0.5 ± 0.5	3.9 ± 0.4***^,a,b^	2.1 ± 0.3^a^	**+**
Central, lateral	0.0 ± 0.0	0.3 ± 0.2	0.3 ± 0.2	0.2 ± 0.2	1.4 ± 0.4	1.1 ± 0.3	
Central, capsular	0.7 ± 0.3	0.9 ± 0.6	0.7 ± 0.3	0.3 ± 0.2	2.4 ± 0.5	2.6 ± 0.5	
Lateral, dorsolateral	1.1 ± 0.4	1.2 ± 0.4	0.7 ± 0.4	1.2 ± 0.4	2.1 ± 0.4	2.7 ± 0.4	
Basolateral, anterior	3.8 ± 0.5	3.3 ± 0.3	3.3 ± 0.9	3.8 ± 0.2	3.9 ± 0.3^†^	6.1 ± 0.4^a^	**−**
Basolateral, posterior	1.2 ± 0.3	0.6 ± 0.3	0.3 ± 0.2	0.8 ± 0.2	1.5 ± 0.2	1.8 ± 0.3	
Basomedial, anterior	1.2 ± 0.3	1.4 ± 0.5	0.7 ± 0.2	1.5 ± 0.5	1.7 ± 0.5	3.2 ± 0.5	
Medial, anterodorsal	4.6 ± 0.6	5.8 ± 0.5	3.0 ± 0.6	4.3 ± 0.3	4.7 ± 0.8	5.9 ± 0.5	
Medial, anteroventral	4.3 ± 0.7	5.2 ± 0.8	2.8 ± 0.6	3.5 ± 0.5	5.4 ± 0.8	5.3 ± 0.8	
Cortical, anterior	6.0 ± 1.4	8.7 ± 1.0	3.3 ± 0.7	5.8 ± 1.6	8.1 ± 0.8	13.4 ± 1.3	
Cortical, posterolateral	4.2 ± 0.8	6.7 ± 1.0	2.5 ± 1.0	3.8 ± 0.9	4.9 ± 0.6	8.4 ± 0.7	
Bregma −2.80							
Central, medial	2.3 ± 0.3	2.4 ± 0.4	1.0 ± 0.5	2.3 ± 0.2	3.7 ± 0.8	1.2 ± 0.2	
Central, lateral	1.3 ± 0.3	1.6 ± 0.4	1.3 ± 0.6	2.2 ± 0.2	2.0 ± 0.2	1.4 ± 0.2	
Central, capsular	1.1 ± 0.4	1.6 ± 0.6	1.0 ± 0.0	1.7 ± 0.4	2.1 ± 0.4	1.8 ± 0.3	
Lateral	2.2 ± 0.3	2.0 ± 0.3	2.0 ± 0.3	1.8 ± 0.6	2.6 ± 0.3	4.0 ± 0.5	
Basolateral, anterior	4.0 ± 0.8	4.5 ± 0.4	3.7 ± 0.6	3.8 ± 0.4	5.1 ± 0.5	5.3 ± 0.3	
Basomedial, anterior	1.1 ± 0.4	1.5 ± 0.4	0.5 ± 0.0	1.0 ± 0.3	1.3 ± 0.3	1.8 ± 0.4	
Medial, anterodorsal	3.2 ± 0.5	4.3 ± 0.4	2.8 ± 0.6	3.8 ± 0.2	4.1 ± 0.2	6.7 ± 0.6	
Medial, posteroventral	4.6 ± 0.8	6.3 ± 0.4	2.7 ± 0.7	3.7 ± 0.4	6.2 ± 0.8	10.4 ± 1.1	
Cortical, anterior	3.9 ± 0.8	7.3 ± 0.5	1.7 ± 0.3	3.7 ± 1.0	4.9 ± 0.6	9.3 ± 0.9	
Cortical, posterolateral	3.5 ± 0.8	4.3 ± 0.6	2.2 ± 1.0	3.2 ± 0.2	4.8 ± 0.5	8.6 ± 0.8	
Bregma −3.30							
Central	2.8 ± 0.5	4.3 ± 0.6	3.5 ± 0.9	2.8 ± 0.8	4.8 ± 0.7	4.7 ± 0.6	
Lateral	1.5 ± 0.2	2.1 ± 0.2	1.7 ± 0.3	2.2 ± 0.2	2.0 ± 0.3***	4.2 ± 0.2^a,b^	**−**
Basolateral, anterior	3.7 ± 0.6	3.8 ± 0.3	3.3 ± 0.4	3.0 ± 0.8	3.2 ± 0.4	4.4 ± 0.5	
Basolateral, posterior	1.2 ± 0.2	1.4 ± 0.1	1.3 ± 0.3	1.5 ± 0.5	1.3 ± 0.2	1.2 ± 0.1	
Basomedial, posterior	2.2 ± 0.5	2.7 ± 0.4	2.3 ± 0.3	2.2 ± 0.3	3.0 ± 0.6	4.3 ± 0.5	
Medial, posterodorsal	1.6 ± 0.4	2.5 ± 0.6	0.8 ± 0.4	0.7 ± 0.4	2.7 ± 0.9	3.5 ± 0.6	
Medial, posteroventral	4.8 ± 0.5	5.5 ± 0.4	1.7 ± 0.4	4.0 ± 0.8	5.3 ± 0.6	7.1 ± 0.5	
Cortical, posterolateral	4.0 ± 0.4	4.4 ± 0.4	2.7 ± 0.4	3.3 ± 1.2	3.6 ± 0.4	6.3 ± 0.6	
Bregma −3.60							
Lateral	1.8 ± 0.3	2.0 ± 0.3	1.2 ± 0.4	1.8 ± 0.2	2.2 ± 0.4	4.4 ± 0.6	
Basolateral, posterior	1.8 ± 0.4	2.1 ± 0.6	1.8 ± 0.4	2.0 ± 0.0	2.9 ± 0.5	2.8 ± 0.3	
Medial, posterodorsal	0.8 ± 0.1	1.3 ± 0.3	0.7 ± 0.2	0.8 ± 0.2	2.2 ± 0.4	2.4 ± 0.4	
Basomedial, posterior	2.5 ± 0.4	2.8 ± 0.5	1.8 ± 0.4	3.3 ± 0.3	2.9 ± 0.6	4.2 ± 0.4	
Cortical, posterolateral	2.5 ± 0.8	3.3 ± 0.6	1.2 ± 0.2	1.7 ± 0.2	3.9 ± 0.5	4.7 ± 0.3	
Cortical, posteromedial	1.9 ± 0.4	4.0 ± 0.6	1.7 ± 0.3	2.2 ± 0.9	3.2 ± 0.5	5.1 ± 0.6	
Medial prefrontal cortex							
Bregma +2.70							
CG area 1	5.6 ± 0.8	6.7 ± 0.5	5.7 ± 0.8	4.8 ± 0.8	5.9 ± 0.4***	10.3 ± 0.5^a,b^	**−**
Prelimbic cortex	5.1 ± 0.8	5.8 ± 0.5	5.8 ± 0.7	5.5 ± 0.9	5.6 ± 0.4	8.3 ± 0.4	
Infralimbic cortex	3.8 ± 0.6	6.0 ± 0.5	4.8 ± 0.4	4.3 ± 1.2	4.6 ± 0.6***	8.8 ± 0.5^a,b^	**−**
Bregma +2.20							
Cingulate cortex area 1	5.8 ± 0.4	5.6 ± 0.8	4.8 ± 0.6	5.3 ± 0.7	3.4 ± 0.6**	7.3 ± 0.7	**−**
Prelimbic cortex	5.9 ± 0.5	5.3 ± 0.3	4.8 ± 0.2	5.2 ± 0.2	4.6 ± 1.0	6.1 ± 0.3	
Infralimbic cortex	4.5 ± 0.3	4.4 ± 0.3	4.0 ± 0.3	4.3 ± 0.2	5.0 ± 0.6	5.3 ± 0.3	

Values are mean ± SEM numbers of c-Fos-positive cells/0.01 mm². Extinction group: C(+)CS HAB (*n*= 7), LAB (*n*= 9). Control groups: C(+)no-CS HAB and LAB (*n*= 5, respectively); C(−)CS HAB and LAB (*n*= 3, respectively). ^†^*P* = 0.07, **P<0.01, ***P<0.001 vs. LAB C(+)CS group. ^a^*P*< 0.01 vs. corresponding C(+)no-CS groups. ^b^*P*< 0.01 vs. corresponding C(−)CS groups. −, reduced c-Fos expression in HAB vs. LAB rats, +, increased c-Fos expression in HAB vs. LAB rats.

#### c-Fos immunohistochemistry

At 2 h after placement of rats into context B, animals were deeply anaesthetized with an overdose of sodium pentobarbital (200 mg/kg) and transcardially perfused with 100 mL of 0.9% saline followed by 100 mL of 4% paraformaldehyde in 0.1 m phosphate-buffered solution (pH 7.4). Brains were then removed and post-fixed at 4°C overnight in 4% paraformaldehyde in phosphate-buffered solution. Coronal sections (50 μm) were cut with a Vibratome (Ted-Pella, Inc., Redding, CA, USA) and collected in Immunobuffer. The sections were processed for c-Fos immunoreactivity as described previously (Singewald *et al.*, 2003). Briefly, sections were incubated for 48 h in a polyclonal primary antibody (sc-52; Santa Cruz Biotechnology, Santa Cruz, CA, USA) diluted (1 : 20 000) in immunobuffer (pH 7.4) comprising 0.1 m NaCl, 5 mm KCl, 8 mm Na_2_HPO_4_, 15 mm NaH_2_PO_4_, 10 mm Tris–HCl, 0.3% Triton X-100 and 0.04% Thimerosal. The rabbit primary antibody was raised against a peptide mapping at the amino terminus of c-Fos p62 of human origin and is not cross-reactive with Fos B, Fra-1 or Fra-2. The sections were then rinsed and placed in a biotinylated goat anti-rabbit secondary antibody (1 : 200; Vector Laboratories, Burlingame, CA, USA) for 24 h. An avidin-biotin-horseradish peroxidase procedure (Vectastain ABC Kit; Vector Laboratories) with 3,3′-diaminobenzidine as the chromogen was used to visualize c-Fos-positive cells. Cells containing a nuclear brown/black reaction product were considered as c-Fos-positive cells. The anatomical localization of c-Fos-positive cells was aided by the use of adjacent Nissl-stained sections and the illustrations in a stereotaxic atlas (Paxinos & Watson, 1998). All cells that were unambiguously distinguishable from background staining were bilaterally counted in each region of interest within a defined area (0.01 mm^2^). This was performed by an observer blind to the experimental groups.

#### Acetylcholinesterase immunohistochemistry

Acetylcholinesterase staining was performed as described previously by Hedreen *et al.* (1985). This procedure aided the identification of subdivisions in the amygdala.

### Statistical analyses

Statistical evaluation (Statistica 7.1, StatSoft, Inc., Tulsa, AR, USA) of conditioning and extinction was performed by either one- or two-way anova and one- or two-way anova with repeated measures. Freezing behaviour in extinction recall was evaluated using Student’s *t*-test. c-Fos data were analysed using two-way anova followed by a Bonferroni multiple comparisons correction post-hoc analysis. Correlation between freezing within extinction and the number of faecal boli or c-Fos-positive cells was performed by using Spearman’s coefficient test. The threshold for statistical significance was set at *P*<0.05.

## Results

### Experiment 1

#### Acquisition of cued fear

**Fig. 1:**
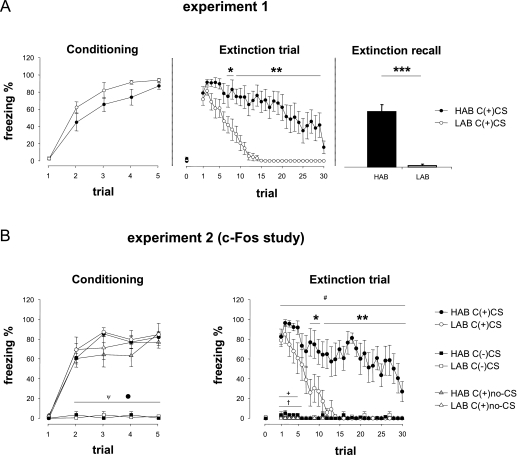
(A) Experiment 1 [C(+)CS groups only]. Freezing responses during the fear conditioning trial did not differ between HAB and LAB rats. After repeated conditioned auditory stimulus presentations during the extinction trial, LAB rats showed a progressive decline in freezing behaviour, whereas freezing in HAB rats remained elevated until the 29th CS presentation. Note that no freezing was observed in all groups of cued-conditioned rats upon exposure to context alone (trial 0). HAB, *n*= 9; LAB, *n*= 10. During extinction recall, HAB rats showed elevated levels of freezing compared with LAB rats. (B) Experiment 2 (c-Fos study). The freezing responses in C(+)CS HAB and LAB rats were similar to those observed in Experiment 1. Non-conditioned animals [C(−)CS groups] did not freeze during the conditioning. Animals of both control groups [C(−)CS and C(+)no-CS groups] did not freeze during the extinction trial. Trials in conditioning (left image), number of CS/US pairings; trials in extinction (right image), number of CS presentation. *P<0.05, **P<0.01, ***P<0.001, HAB C(+)CS vs. LAB C(+)CS group; ^#^*P*<0.01, HAB C(+)CS vs. HAB C(−)CS and HAB C(+)no-CS groups; ^•^*P*<0.01, LAB C(+) vs. LAB C(−) group; ^ψ^*P*>0.01, HAB C(+) vs. HAB C(−) group; ^+^*P*>0.01, LAB C(+)CS vs. LAB C(−)CS group; ^†^*P*>0.01, LAB C(+)CS vs. LAB C(+)no-CS group. Extinction groups: C(+)CS HAB (*n*= 7) and LAB (*n*= 9); Control groups: C(+)no-CS HAB and LAB (*n*= 5, respectively), and C(−)CS HAB and LAB (*n*= 3, respectively).

During the conditioning trial (day 1) following repeated CS/US pairing, the percentage of freezing behaviour increased continuously in both HAB and LAB rats from 0% during the first CS to about 80–90% within the fifth CS presentation. Trait anxiety did not affect acquisition of fear as both lines froze similarly during the whole acquisition trial [interaction (time × group), *F*_4,68_ = 0.937, *P*=0.448] (Fig. 1A).

#### Extinction trial

In the extinction trial (day 2), no freezing behaviour was displayed during the first 5 min in the extinction chamber (prior CS presentation, trial 0), indicating excellent discrimination of context B and A. Rats of both lines showed similarly pronounced expression of fear (around 80% freezing) during the first CS presentation. After repeated CS presentation, a rapid decrease in freezing behaviour was observed in LAB rats down to very low levels (5%) after 13 CS presentations, whereas HAB rats continued to display high levels of freezing across the course of the test session (70% freezing after 13 CS, 40% after 25 CS) [interaction (time × group), *F*_30,15_=2.802, *P*<0.001]. After 30 CS presentations, low freezing levels (10–15%) were also observed in the HAB line, which no longer differed statistically from the LAB line (Fig. 1A).

#### Extinction recall

In the extinction recall session (day 3), animals did not show freezing within the 5 min habituation time, supporting the good context discrimination (see above). During the single CS presentation, HAB rats showed a high freezing level (63%) compared with LAB rats that displayed almost no freezing (∼2%) [*H*(1, *N*=19) = 15.0882, *P*<0.001].

#### Defaecation

The HAB and LAB rats showed similar defaecation during the conditioning trial (HAB, 3.9 ± 0.6; LAB, 2.8 ± 0.5) (*P*>0.05). In the extinction trial, however, HAB rats produced a larger number of faecal boli compared with LAB rats (HAB, 3.8 ± 0.3; LAB, 1.9 ± 0.6) (*P*<0.05). Correlation analysis between freezing and the amount of faecal boli revealed an interdependency of both measures (*R*=0.6430).

### Experiment 2

#### Behaviour

As expected, rats of both conditioned groups [C(+)CS and C(+)no-CS (day 1)] showed pronounced freezing responses in the conditioning trial on day 1, [interaction (line × group × time), *F*_8,104_=8.617, *P*<0.001] whereas no freezing was observed in rats of the C(−)CS group. During the whole extinction trial on day 2, rats of both control groups [C(+)no-CS and C(−)CS] did not freeze and thus differed significantly from rats of the C(+)CS groups, which showed pronounced freezing in response to CS presentation. Repeated CS presentations revealed essentially the same differences in the attenuation of freezing between HAB and LAB rats, as observed in Experiment 1. The variation seems higher but this is probably due to the lower *n*-numbers of experimental animals [interaction (line × group × time), *F*_60,78_=1.392, *P*<0.05] (see Fig. 1B).

#### c-Fos expression in the amygdala and mPFC

The c-Fos expression in subregions of the amygdala and mPFC of HAB and LAB rats was evaluated in all three experimental groups [C(+)CS, C(+)no-CS and C(−)CS]. There was a significant line × group interaction for the number of c-Fos-positive cells in the lateral nucleus of the amygdala (LA) (−3.30 mm Bregma, *F*_2,32_ = 3.86, *P*<0.01), basolateral nucleus of the amygdala (BLA) (−2.30 mm Bregma, *F*_2,32_ = 3.59, *P*<0.05), medial part of the central nucleus of the amygdala (CeM) (−2.30 mm Bregma, *F*_2,32_ = 9.87, *P*<0.01), infralimbic cortex (IL) (+2.70 mm Bregma, *F*_2,32_ = 7.25, *P*<0.01) and cingulate cortex (CG) (area 1) (+2.70 mm Bregma, *F*_2,32_ = 10.94, *P*<0.01; +2.20 mm Bregma, *F*_2,32_ = 4.11, *P*<0.05). *Post-hoc* statistical analysis revealed the following results (see below; Table 1).

#### c-Fos expression in HAB and LAB control groups

Low to moderate c-Fos expression was detected in different parts of the amygdala and mPFC of rats of both control groups [C(+)no-CS and C(−)CS]. Quantification of the c-Fos expression revealed no differences between HAB and LAB rats in either the C(+)no-CS or C(−)CS group.

#### c-Fos expression in HAB and LAB rats of the extinction group

The LAB rats of the extinction group [C(+)CS] displayed significantly more c-Fos-positive cells in the LA (*P*<0.001), BLA (*P*<0.05), CeM (*P*<0.05), IL (*P*<0.05) and CG (area 1) (*P*<0.01) compared with the LAB rats of the C(+)no-CS control group, and in the LA (*P*<0.01), IL (*P*<0.001) and CG (area 1) (*P*<0.001) compared with the LAB rats of the C(−)CS control group. C(+)CS HAB rats showed increased c-Fos expression in the CeM (*P*<0.001) in comparison to HAB rats of both control groups. A correlation analysis between freezing behaviour and c-Fos expression revealed a highly significant correlation in the CeM (*R*=0.95) and moderate correlations in the BLA (*R* = 0.63), LA (*R* = 0.58), CG (area 1) (*R* = 0.56) and IL (*R* = 0.48).

**Fig. 3:**
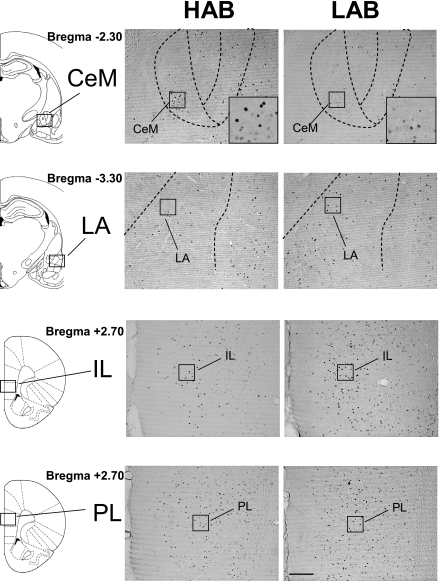
Bright-field photomicrographs of representative sections showing significant differences in c-Fos expression within the medial part of the CeM and LA, and the IL in HAB and LAB rats after the extinction trial [C(+)CS groups only]. High-power photomicrograph showing examples of c-Fos-positive cells in the CeM. The prelimbic cortex (PL) is shown as an example of similar c-Fos responses in both lines. The selected areas are depicted in the schematic drawings (adapted from Paxinos & Watson, 1998). Scale bar, 200 μm.

Most importantly, however, differential c-Fos responses to CS presentations in the extinction trial were observed between HAB C(+)CS and LAB C(+)CS animals in specific subregions of the amygdala and mPFC (Fig. 2, Table 1). In the CeM, HAB rats showed a facilitated activation, as the number of c-Fos-positive cells was increased in this brain area compared with LAB rats (*P*<0.001) (Fig. 3). In contrast, HAB rats exhibited an attenuated c-Fos response in the LA (*P*<0.001) (Fig. 3) and BLA, although in the latter brain area the *post-hoc* analysis failed to reach statistical significance (Bregma −2.30, *P* = 0.07). These differences in c-Fos expression were evident at particular rostro-caudal brain levels (Table 1). In the mPFC, a reduced c-Fos response to repeated CS presentations was found in the IL (*P*<0.001) of HAB rats as well as in the CG (area 1) at both levels quantified (+2.20 mm Bregma, *P*<0.05; +2.70 mm Bregma, *P*<0.001) (Fig. 3).

## Discussion

By using a classical cued fear conditioning paradigm, we found for the first time that HAB rats with a genetic predisposition to hyperanxiety (for review, see e.g. Landgraf *et al.*, 2007) show evidence of impaired extinction of conditioned fear, whereas no differences in fear acquisition were observed when compared with LAB rats. The reduced extinction learning ability of HAB rats was accompanied by an altered neuronal activation pattern in subregions of the amygdala and mPFC, both brain structures that are known to play a key role in fear extinction processes (see Introduction). Notably, we revealed hypoactivation in the mPFC, in particular in the IL and CG, but hyperactivation of the CeM, which is considered the main output region of amygdala projections that mediate specific behavioural and autonomic fear responses (LeDoux *et al.*, 1988; Pare *et al.*, 2004; Maren, 2005). Conversely, the rapid extinction in LAB rats was associated with enhanced activation in the mPFC, whereas activation of the central amygdala was attenuated, reflecting the inhibition of the behavioural conditioned fear response indicated by decreased freezing in these animals. Hence, a dual regulatory capacity, dependent upon the genetic predisposition to trait anxiety, exists in prefrontal-amygdala areas.

### Conditioned fear behaviour

The major interest of the present study was to test whether HAB rats may represent a psychopathologically relevant animal model of impaired fear memory extinction, thus mimicking a core problem of various anxiety disorders (see Introduction). Therefore, we aimed to achieve considerable freezing responses at the beginning of the extinction trial in both rat lines by using a relatively strong conditioning paradigm including five pairings. We also applied temporally massed CS presentations on day 2 to generate intense fear extinction (Cain *et al.*, 2003). During all five CS/US pairings in the conditioning trial, HAB and LAB rats showed equal freezing responses that finally reached levels of approximately 80–90%. The finding of similar fear acquisition in both groups suggests that the selective bidirectional breeding procedure *per se* had no influence on their general abilities to build fear memories in the fear conditioning paradigm. In contrast, in other less aversive learning tasks, including the social discrimination test and visuospatial tasks such as the modified holeboard test, HAB rats displayed better learning and memory abilities, and signs of higher stress susceptibility than their low anxiety counterparts (for review, see Landgraf & Wigger, 2002; Magich Marina MPI Munich, unpublished observation). It is likely that each of these tests may induce a distinct type of aversiveness, which could result in the formation of different memories stored in distinct neuronal structures (LeDoux, 2000; Maren, 2005; Phelps & LeDoux, 2005). However, the conditions used in the present study may have masked possible differences in fear acquisition between the lines. Using a low current intensity (e.g. 0.3 mA) and a lower number of pairings may reveal such differences. However, the conditioning process was not the main focus of the present study. In addition to the freezing response, the defaecation during the acquisition trial was also similar in HAB and LAB rats. As enhanced defaecation has been associated with increased emotional states in rodents (Vanderwolf *et al.*, 1988; Sarbadhikari *et al.*, 1996), this further supports the finding that the foot-shock/tone pairings induced similar fear in both lines. Probably due to their methodological heterogeneity, human studies in anxiety disorder patients yielded mixed results concerning fear acquisition (Mineka & Oehlberg, 2008), although a previous meta-analysis revealed evidence of a stronger acquisition of conditioned fear in anxiety patients (Lissek *et al.*, 2005).

Freezing (indicative of fear expression) in response to the first two CS presentations on day 2 was at a high level similar to that after conditioning on day 1 and did not differ between the lines. This indicates that the consolidation of fear memory was similarly strong in HAB and LAB rats under the conditions used. Strikingly, however, a marked line difference was revealed in response to further non-reinforced CS presentations during the extinction trial. While a rapid decrease in freezing behaviour was observed in LAB rats, HAB rats showed a marked delay in fear extinction indicated by sustained freezing behaviour. Preliminary studies (Muigg and Hetzenauer, unpublished data) in unselected Wistar rats indicated that the decline in freezing during the fear extinction trial is intermediate between that observed in HAB and LAB rats. Higher freezing levels in HAB compared with LAB rats were also observed in an extinction recall test assessed 24 h later. This is in accordance with human data summarized in the meta-analysis of Lissek *et al.* (2005), in which patients with anxiety showed persistently elevated levels of conditioned fear responses during extinction training as compared with normal controls. Interestingly and in contrast to the better learning abilities in the less aversive tests mentioned above, HAB rats seem to have clear deficits in the extinction of fear memory, which is considered to be a form of new, inhibitory learning that counteracts the expression of the excitatory conditioned responses (Pavlov, 1927; Konorski, 1948; for recent review see Myers & Davis, 2007; Quirk & Mueller, 2008).

In the present study we also found that defaecation during the extinction trial mirrored the freezing responses, as the number of faecal boli shed was considerably higher in HAB vs. LAB rats. Although some studies point to a rather loose association of defaecation with other anxiety measures (Courvoisier *et al.*, 1996; Ramos *et al.*, 1997, 1998; Henderson *et al.*, 2004), the present results indicate an association with fear responses such as freezing (see also Paterson *et al.*, 2001; Pietersen *et al.*, 2006).

Taken together, the behavioural data of HAB animals characterized by enhanced trait anxiety indeed show deficits in the extinction of acquired fear memory, closely mirroring findings in anxiety patients.

### Altered prefrontal-amygdala activation profile in HAB rats

Using c-Fos mapping we revealed a different prefrontal-amygdala activation profile in C(+)CS HAB vs. LAB rats in response to repeated CS presentations in the extinction trial, whereas no differential c-Fos responses were observed in the control groups [C(+)no-CS and C(−)CS]. Specifically, c-Fos responses in HAB rats were reduced in the IL and CG, as well as in the LA and BLA, whereas they were increased in the CeM. Given that high and low levels of freezing were observed during extinction in C(+)CS HAB and LAB rats, respectively, potential differences in the c-Fos response should reflect the behavioural differences during the extinction trial rather than initial fear retrieval, which was similar at the onset of CS presentations (see also Hefner *et al.*, 2008). As fear memory retrieval is necessary for extinction learning, contributions of both phenomena to the c-Fos response must be assumed.

#### Amygdala

Although there is growing evidence that fear conditioning and fear extinction are independent forms of learning that are mediated by partially dissociable neural mechanisms, the amygdala seems to be important in both (Kamprath & Wotjak, 2004; Myers & Davis, 2007; Quirk & Mueller, 2008). Extinction of conditioned fear is thought to occur through specific intra-amygdaloid circuitries involving, in particular, the LA/BLA and central amygdala interacting with additional intra- and extra-amygdaloid areas including, most importantly, the mPFC (e.g. Pare *et al.*, 2004; [Bibr b42][Bibr b69]; Sotres-Bayon *et al.*, 2006; Herry *et al.*, 2008). A key finding of the present study was that the sustained fear response reflecting enhanced resistance to extinction in HAB rats was associated with a facilitated c-Fos response in the CeM compared with fast-extinguishing LAB rats, whereas in the LA and BLA, more recently implicated in extinction learning (Herry *et al.*, 2006; Sotres-Bayon *et al.*, 2007), attenuated c-Fos responses were observed in HAB rats. Thus, the present results point to altered neuronal processing in key areas of the intra-amygdaloid fear circuitry that are thought to play a critical role in the extinction of learned fear. It will be necessary in future studies to perform c-Fos double-labelling experiments with markers for GABAergic and glutamatergic neurons, for example, to differentiate between (pheno)types of activated cells in the amygdala and mPFC (see below). Interestingly, Kubota *et al.* (2004) also revealed that the number of c-Fos-positive cells was increased in the central amygdala and reduced in the BLA and LA after an extinction trial in anxious Fyn(−/−) mice compared with low anxious Fyn(+/−) mice. Unfortunately, in the study of Kubota *et al.* (2004) the central amygdala was not divided into subdivisions. The CeM subdivision is suggested to be the main output region for amygdala projections that mediate specific fear responses, including freezing and autonomic responses (LeDoux *et al.*, 1988). Indeed, the enhanced c-Fos responses in the CeM of HAB rats was correlated with the higher freezing noted in HAB vs. LAB rats, which is in accordance with results in the central amygdala after fear extinction in mice with impaired extinction (Hefner *et al.*, 2008).

Notably, the differential neuronal activity found in restricted amygdala subdivisions was apparent at specific levels of the anterior/posterior axis, indicating that functionally distinct neuronal populations were differentially affected. Indeed, it is known that the amygdaloid nuclei differ in cytoarchitectonic, chemoarchitectonic and connectional ways. Consistent with anatomical data, various nuclei or nuclear groups also differ functionally (Pitkänen, 2000). This gives rise to the notion that specific subpopulations within certain amygdaloid nuclei at specific rostro-caudal levels are involved in the mediation of the differential fear extinction behaviour observed in HAB compared with LAB rats.

#### mPFC

The mPFC provides an interface between limbic and cortical structures (Groenewegen & Uylings, 2000). An important role proposed for the mPFC in cued fear extinction is the regulation of fear expression via inhibition of the amygdala, although details of this regulation are still a matter of debate (for recent review, see Maren, 2005; Sotres-Bayon *et al.*, 2006; Myers & Davis, 2007; Quirk & Mueller, 2008). Within the mPFC, the IL seems to be particularly important for extinction learning (Quirk *et al.*, 2000; Milad & Quirk, 2002; Milad *et al.*, 2004; Santini *et al.*, 2004; Berretta *et al.*, 2005). Evidence for this comes from various lesion, infusion and stimulation studies that were reviewed recently by Quirk & Mueller (2008).

In the present study, we found an attenuated c-Fos response after repeated CS presentations in the IL and CG (area 1) but not in the prelimbic cortex of HAB compared with LAB rats. Hypoactivation in the CG of HAB rats was previously also found in response to a range of emotional challenges, including exposure to the open arm of an elevated plus maze, open field test and social defeat (Salome *et al.*, 2004; Frank *et al.*, 2006; for review, see Singewald, 2007). However, reduced activation of the IL was not observed in response to any of these behavioural challenges. This indicates that certain aspects of dysfunctional cortico-limbic activation in HAB rats are part of a general feature associated with the enhanced trait anxiety in these animals and can be revealed by different challenging paradigms. Although evidence of an implication of the CG in extinction mechanisms has been observed particularly in human studies so far (e.g. Phelps *et al.*, 2004; Milad *et al.*, 2006; Bryant *et al.*, 2008), the implication of IL neurons in the inhibition of conditioned fear after extinction is well established (Milad *et al.*, 2004; Quirk *et al.*, 2006). Ventromedial prefrontal units fire in relation to the generation of extinction (Milad & Quirk, 2002) and extinction increases burst firing (Burgos-Robles *et al.*, 2007; Santini *et al.*, 2008) and has been shown to potentiate IL activity (Milad & Quirk, 2002; Herry & Mons, 2004). Considering the evidence of an inhibitory IL/amygdala pathway (Quirk *et al.*, 2003), the reduced IL activation in HAB rats may contribute to the observed hyperactivation of neurons in the CeM. In other words, the rapidly extinguishing LAB rats showed enhanced activation of the IL as compared with the poorly extinguishing HAB rats. Also in humans, the extinction of an aversively conditioned reflex is accompanied by increased activity in the ventromedial prefrontal cortex (Phelps *et al.*, 2004; Milad *et al.*, 2005; Kalisch *et al.*, 2006). Further support for this interpretation comes from a metabolic mapping study with radiolabelled fluorodeoxyglucose in mice (Barrett *et al.*, 2003). One major finding was that successful extinction is associated with elevated activity in the IL and CG (area 1), but not in the prelimbic cortex, and reduced activation of central amygdala neurons (Barrett *et al.*, 2003). Although this method has a lower spatial resolution, the observed pattern of activation is very similar to that observed in the rapidly extinguishing LAB rats. Along similar lines, low freezing responses observed in a within-session extinction trial were accompanied by enhanced c-Fos response in mPFC areas including the IL (Morrow *et al.*, 1999). This was also found in extinguished vs. non-extinguished Sprague-Drawley rats (Santini *et al.*, 2004), unconditioned vs. conditioned C57Bl/6 mice (Herry & Mons, 2004) and extinguishing (C57Bl6) vs. non-extinguishing (129S1) mice (Hefner *et al.*, 2008).

### Conclusions

Taken together, we demonstrate for the first time that HAB rats, a psychopathological animal model of increased trait anxiety, show considerable deficits in the ability to extinguish learned fear. The sustained fear response during repeated CS presentation in HAB rats as compared with rapidly extinguishing LAB rats was associated with overactivation in the CeM, whereas parts of the mPFC (IL and CG) as well as the lateral and basolateral subnuclei of the amygdala showed attenuated activation. These findings provide evidence that impairment of neuronal processing in these key areas of extinction pathways contributes to aberrant extinction abilities in HAB rats. In particular, the lack of adequate mPFC activation, which may contribute to hyperactivation of the central nucleus of the amygdala and thus sustained freezing responses, seems to be involved in the mediation of the observed resistance to extinction and impaired recall of extinction. Support for the hypothesis of amygdala hyper-responsivity to fear-related stimuli, with a concomitant lack of ‘top-down’ control (e.g. Milad *et al.*, 2006; Rauch *et al.*, 2006; Akirav & Maroun, 2007), comes from different neuroimaging studies demonstrating a hyporesponsive mPFC in normal subjects who fail to recall extinction memory (Milad *et al.*, 2007) and in post-traumatic stress disorder patients exposed to trauma-related stimuli (Bremner *et al.*, 1999; Liberzon *et al.*, 1999; Shin *et al.*, 1999, 2004; Rauch *et al.*, 2000). The inability to extinguish fear memories, often in combination with a bias toward favouring the selective processing of threat cues and explicit memory for threat, is a major problem not only in post-traumatic stress disorder but also in other anxiety disorders, including phobias, panic and obsessive-compulsive disorders. The elucidation of how the brain mediates the extinction process should lead to better therapies for these disorders. The HAB/LAB model, therefore, may represent a relevant tool to further investigate brain mechanisms of impaired fear extinction and to test pharmacological approaches to facilitate this process.

## Abbreviations

BLA: basolateral nucleus of the amygdala
CeM: medial part of the central nucleus of the amygdala
CG: cingulate cortex
CS: conditioned stimulus
HAB: high anxiety-related behaviour
IL: infralimbic cortex
LA: lateral nucleus of the amygdala
LAB: low anxiety-related behaviour
mPFC: medial prefrontal cortex
US: unconditioned stimulus
